# Preceding diagnoses to young adult bipolar disorder and schizophrenia in a nationwide study

**DOI:** 10.1186/1471-244X-13-343

**Published:** 2013-12-21

**Authors:** Søren Martin Andersen, Anne Randers, Christina Mohr Jensen, Charlotte Bisgaard, Hans-Christoph Steinhausen

**Affiliations:** 1 Department of Child and Adolescent Psychiatry, Aalborg Psychiatric Hospital, Aalborg University Hospital, Mølleparkvej 10, DK 9000 Aalborg, Denmark; 2 Research Unit for Child and Adolescent Psychiatry, Aalborg Psychiatric Hospital, Aalborg University Hospital, Aalborg, Denmark; 3 Clinical Psychology and Epidemiology, Institute of Psychology, University of Basel, Basel, Switzerland; 4 Department of Child and Adolescent Psychiatry, University of Zurich, Zurich, Switzerland

**Keywords:** Follow-back study, Preceding diagnoses, Bipolar disorders, Schizophrenia

## Abstract

**Background:**

The aim of this comparative study was to investigate the type and frequency of diagnoses preceding adult bipolar disorder (BD) and schizophrenia (SZ).

**Methods:**

A follow-back study of all preceding diagnoses in all patients aged 21–34 years with a primary, first time diagnosis of BD (N = 784) or SZ (N = 1667) in 2008 to 2010. Data were taken from the Danish Psychiatric Central Research Register (DPCRR) including ICD-10 and ICD-8 diagnoses.

**Results:**

The numbers of patients with any preceding diagnoses amounted to 69.3% in BD and 76.6% in SZ with affective disorders (excluding BD) being the most frequent preceding diagnosis (46.6 vs. 28.0%), followed by psychoses (PSY) other than SZ (14.2 vs. 41.5%, p < .001), and substance use disorders (SUD) (16.1 vs. 26.9%, p < .001). Reactions to severe stress were equally frequent in both samples (26.3 vs. 26.6%) as were personality disorders (21.8 vs. 22.4%) and ADHD (4.2 vs. 3.5%), whereas rates of conduct disorders (1.7 vs. 3.1%) were rather low in both samples. Very few of the preceding diagnoses had their onset in childhood and adolescence. Overall patients with SZ had a minor but statistically significant earlier onset of any psychiatric disorder compared to BD (mean age: 23.3 vs. 22.5, p < .001). Regression analyses indicated that BD was associated with an increased risk of having experienced preceding affective disorders and ADHD, while SZ was associated with an increased risk of preceding substance use disorders, psychosis, anxiety disorders, and personality disorders.

**Conclusions:**

Specific developmental trajectories of preceding disorders were delineated for BD and SZ with affective disorders being more specific for BD and both SUD and PSY more specific to SZ. There are different patterns of vulnerability in terms of preceding diagnosis in young adults with BD and SZ.

## Background

Studies in developmental psychopathology based on longitudinal-epidemiological approaches have shown that adult mental disorders are often preceded by various psychiatric disorders originating in childhood or adolescence. For instance, the longitudinal study of the Dunedin birth cohort in New Zealand found that 74% of all adult psychiatric patients received a diagnosis before the age of 18 [1]. However, most of the existing literature failed to establish a clear developmental perspective on diagnostic shifts and continuities in the course of various psychiatric disorders from adolescence to adulthood. Many studies report lifetime prevalence rates of various diagnoses in specific patient populations such as patients with Bipolar Disorder (BD) or Schizophrenia (SZ), but fail to distinguish which disorders occurred prior to, during, and following the onset of the specific disorder. It should be noted, however, that a clear distinction between concurrent comorbidity and sequential comorbidity [2] is vital for a better understanding of the course of psychiatric disorders as patients develop from childhood through adolescence to adulthood.

The following sketch of the literature will briefly summarize studies on the sequential comorbidity as well as studies investigating lifetime comorbidity in BD and SZ. This brief review of preceding disorders will firstly focus on the so-called disruptive or externalizing disorders, the internalizing disorders will then be considered, and it will conclude with substance use disorders and psychoses.

In the recent past, the most intensive debate in this research domain has revolved around the issue of whether or not Attention-Deficit/Hyperactivity Disorder (ADHD) is a frequent misdiagnosis of early signs of BD, or whether it is only comorbid to BD [3]. In the Oregon Adolescent Depression Project (OADP) 11% of adolescents with BD also had ADHD and 3.8% of adolescents with ADHD also had BD [4]. Furthermore, in a large adult sample 19.4% of those with ADHD also had BD and 21% of those with BD also had ADHD [5]. However, conflicting comorbidity rates were found in various cross-sectional community studies [4-8] and due to low rates of BD in these studies, the lack of a correlation between ADHD and BD should be interpreted with caution [9]. Furthermore, multiple studies have documented a high familial load of BD in families with ADHD and vice versa [10]. In contrast, a recent review of all prospective longitudinal studies on the offspring of parents with BD showed that childhood ADHD was not a reliable predictor of the development of BD [11]. Thus, there are conflicting findings on the co-occurrence of these two disorders.

Compared to BD, the debate on an association of ADHD with SZ has been less intensive, but some studies indicate that children and adolescents with ADHD are also at increased risk of developing SZ compared to the general population [12,13]. In a Danish register based study, 3.8% of patients with ADHD developed SZ when followed to the mean age of 31 years. In this study, childhood ADHD cases were compared to non- childhood ADHD controls and the study identified an increased risk of SZ equal to a relative risk of 4.3 (95% CI 1.9-8.57) [13]. In addition, the findings from the Dunedin birth cohort study indicate that ADHD for some patients precede schizophreniform disorders [1]. This study found that ADHD in childhood/adolescence was associated with adult schizophreniform disorders equal to an odds ratio of 4.5 (CI 95% 1.8-11.0) compared to cases who did not have the disorder in adulthood [1].

In addition to ADHD, other childhood disruptive behaviours such as conduct disorder (CD) and oppositional defiant disorder (ODD) have been found in the history of patients with either BD or SZ. Adult patients with BD have been found to have a history of 30% of disruptive behaviour disorders compared to only 5% in non-psychiatric controls [14]. Furthermore, a prospective community study found that patients with adult BD had a significantly increased frequency of preceding disruptive behaviour disorders when compared to community controls (42.9% vs. 11%) [15]. Aggressive behaviours have also been noted as precursors for SZ in adulthood. As demonstrated in a large community based study, childhood or adolescent conduct problems predicted an increased risk for SZ in adulthood [17]. In another study on SZ based on 248 adult males, CD with onset before the age of 15 was comorbid to SZ in 21.3% of the sample. Furthermore, in this study CD was associated with an earlier onset of SZ and overall a more severe course [16]. However, conduct problems have been shown to precede various adult psychiatric disorders and are, therefore, not specific risk-markers for developing either BD or SZ [1,17].

Further studies have dealt with anxiety disorders preceding either BD or SZ. Compared to non-psychiatric controls, adult patients with BD have been found to have an increased frequency of anxiety disorders prior to their BD diagnosis (42% vs. 5%) [14]. An increased risk of adolescent anxiety disorders in patients with BD has been also found in another study comparing adults with BD with community controls (57.1% vs. 6.0%) [15]. However, regardless of the often observed anxiety disorders, the Dunedin study failed to find an increased frequency of childhood anxiety in patients with mania compared to clinical controls [1]. Anxiety disorders are also frequent concurrent and preceding comorbidities in SZ. A recent meta-analysis of the lifetime and one-year prevalence rates of anxiety disorders in SZ found that 38.3% of patients with SZ fulfilled criteria for at least one co-morbid anxiety disorder [18]. Finally, data from a high-risk study indicated that social anxiety was one of the factors associated with conversion to SZ [19].

In addition, various studies have shown that depressive disorders often precede the onset of BD. The frequency of adolescent depression in patients with adult BD in a US community-based study was 28.6% compared to 7.0% in the community controls [15]. In the Dunedin study, patients with adult mania compared to clinical controls had an increased frequency of juvenile depression equal to an adjusted odds ratio of 3.3 (CI 95% 1.2-9.2) [1]. Juvenile depression was also associated with schizophreniform disorders in adulthood equal to an odds ratio of 7.4 (CI 95% 3.6-16.1) compared to patients who did not develop the disorder [1]. As with anxiety disorders, depressive disorders are frequent in both populations of patients with SZ and BD with the risk of depression exceeding the risk found in the general population [20].

Furthermore, there is some evidence that substance use disorders (SUD) frequently precede BD and mania [21,22]. One study investigating the sequence of SUD in first-episode bipolar I patients found that 45.2% met criteria for SUD [21]. Another community based follow-up study found that cannabis use predicted an increased risk for later mania, but also that mania did not predict the onset of cannabis use suggesting that substance use can act as a trigger of BD behaviours [22]. This study found that cannabis use increased the risk of mania onset (OR: 2.5 CI 95% 1.4-4.6) after controlling for a range of socio-demographic factors and psychotic symptoms [22]. Studies have pointed to an association of preceding SUD and SZ with a predominance of alcohol and cannabis use before the onset of SZ [23,24] but the role of cannabis use in the aetiology of SZ is still unclear [25].

Finally, psychosis may precede both BD and SZ. One study following-up patients with first-episode psychosis found that up to 12% of cases with non-affective psychosis later developed BD [26]. Furthermore, an Italian study found that among a population of 1081 first-episode BD patients, 7.96% had preceding psychosis [27]. In a large register-based study from Sweden, the authors found that among patients presenting in psychiatry with a first-episode of psychosis, 5% were diagnosed with BD during a 5-year follow-up and 18% were diagnosed with SZ [28]. Another prospective follow-up study of patients presenting with first-episode psychosis found that after a follow-up period of 10 years, 50% had developed SZ and 16% had developed BD [29]. This finding suggests that psychosis develops into SZ more often than BD, but that conversion from psychosis to BD is not infrequent.

### Aims of the study

In the present study, we examined whether the patterns of preceding psychiatric disorders varied in patients diagnosed with BD or SZ. This was done by studying the frequency of lifetime preceding diagnoses in two nationwide groups of patients diagnosed with BD or SZ for the first time at the age of 21–34 years. In addition to studying the much debated association of BD and ADHD we extended the focus of research also to SZ and included further diagnoses expected to be common clinical precursors to both BD and SZ, namely, SUD, psychoses other than SZ, affective disorders, anxiety disorders, reaction to stress, personality disorders, and CD. Apart from estimating lifetime prevalence of preceding psychiatric disorders in the patient population, we were also interested in identifying prevalence rates of these disorders in childhood and adolescence.

## Methods

### Procedure

We used data from the Danish Psychiatric Central Research Register (DPCRR) and the Danish Central Civil Registration Register (DCR) in the present study. Registry studies are made possible in Denmark, since all inhabitants in Denmark are given a unique personal identification number at birth allowing the identification of a given persons contacts within the health care system across time. The Danish society has enjoyed a well-fare model for decades where all inhabitants get free and accessible treatment in both somatic and psychiatric treatment facilities in the public sector. DPCRR data is built on the activities in these Danish psychiatric public hospitals, thus making registry data on health care a representative and nationwide source of data on the health of the Danish population. The DPCRR covers all contacts with the mental health care system along with all psychiatric diagnoses, the date, and the place of the diagnosis [30]. The DCR provided information on ID-number, sex and date of birth. The study was approved by the Danish Data Protection Agency, National Board of Health and Statistics Denmark.

The current study was designed as a retrospective longitudinal registry-based case–control study. Cases and controls consisted of patients diagnosed for the first time with BD (F31 in ICD-10) or SZ (F20 in ICD-10) in Denmark in young adulthood. All patients who were diagnosed solely in the emergency wards were excluded from the analyses to strengthen the validity of the diagnoses. Cases and controls in the study were selected if they were diagnosed at the age of 21–34 years in 2008–2010. This limitation on age and year of diagnosis was chosen to make the groups as homogenous as possible regarding the age at onset of the disorders.

Patients were followed back from the date before their first diagnosis of either BD or SZ to their time of birth to calculate life time prevalence rates of mental disorders. The decision to follow patients from the date preceding the first diagnosis of BD and SZ was made to focus specifically on the preceding psychiatric course so that preceding and concurrent rates of comorbidity were not mixed. Because the DPCRR has collected data on activities in the public health care system of Denmark since 1969, the registry has complete data covering all of the patients’ lifetime. A total of N = 797 patients were diagnosed with BD and N = 1,680 with SZ using these in- and exclusion criteria. In 13 cases, the onset of BD and SZ co-occurred; these cases were excluded leaving N = 784 patients in the sample of BD patients and N = 1,667 patients in the SZ sample.

We followed the cohort during both the ICD-8 and the ICD-10 period in Denmark based on DPCRR data. Before 1994, the ICD-8 represented the official nosology in Denmark. In 1994, the ICD-10 was introduced as the official classification system. Although before 1995 only inpatient contacts were registered, the data was included to obtain the most complete account for all the years in the patients’ lives.

Preceding diagnoses that were considered for analysis in the present study included *SUD* (303, 304 in ICD-8; F10 in ICD-10), *psychosis other than SZ* (F21-29 in ICD-10), *affective disorders other than BD* (296.0, 300.4 in ICD-8; F32, F33, F34.1, F92.0 in ICD-10), *anxiety disorders* (300.0, 300.2 in ICD-8; F40, F41, F93 in ICD-10), *reactions to severe stress* (307, 308.4 in ICD-8; F43 in ICD-10), *personality disorders* (301 in ICD-8; F60 in ICD-10), *ADHD* (308.3 in ICD-8; F90 in ICD-10), and *CD* (308.1, 308.1-2 in ICD-8; F90.1, F91, F92 in ICD-10).

### Statistical analyses

Data on preceding diagnoses were used to calculate life-time prevalence rates of psychiatric disorders in the two groups. Chi-square tests were used to test for differences of the preceding diagnoses in the BD and SZ sample. Mann–Whitney U-tests were employed for the analysis of age at diagnosis of the preceding disorders because data was not normally distributed. To investigate the effect of sex on the risk of having the various preceding diagnoses in the population a series of binary logistic regression analyses were carried out. In these analyses the outcome was specified as the preceding diagnosis of interest and the covariate in the model was sex (females as reference group) and analyses were controlled for age. Since we suspected that year of birth was strongly related to the risk of having a preceding diagnosis of e.g. ADHD we carried out a series of Cox regression analyses with the various preceding diagnoses as the events (outcome), time being the time from birth to the first time diagnosis of the specific preceding disorder and censoring occurring at the first time diagnosis of BD/SZ. This was done to control for the variation in follow-up time experienced by patients. The variable for year of birth included in the model was dichotomized such that patients were categorized as born before or after 1980. Patients born before 1980 served as the reference group in these analyses and the models were controlled for sex. To evaluate if any of the lifetime preceding disorders predicted a higher risk of either BD or SZ crude and age/sex-adjusted odd ratios were calculated using binary logistic regression analyses. In these analyses BD/SZ were the outcome and the preceding diagnoses, sex and age the co-variates. All analyses were done using IBM SPSS statistics 19^th^ edition.

## Results

**Figure 1:**
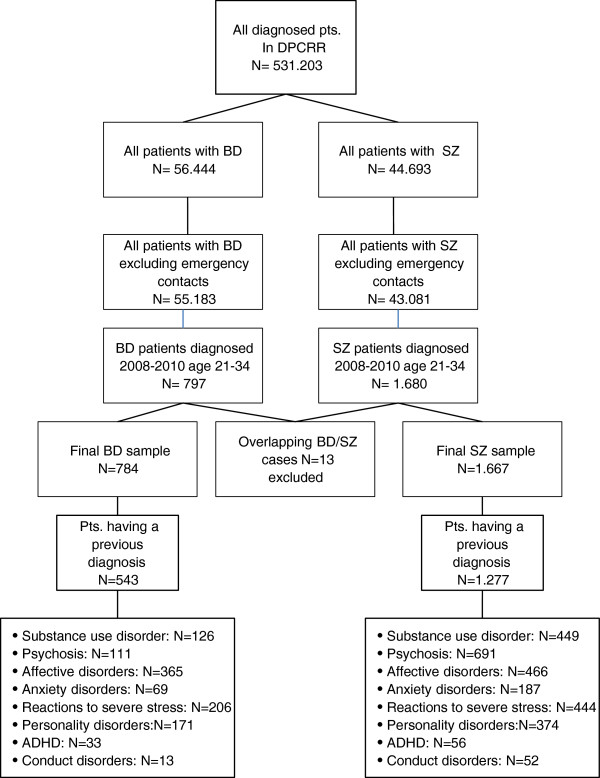
Flowchart.

A flow-chart of the identification of the patients in the present study is provided in Figure 1. There were significantly different sex distributions in the samples with more females in the BD sample (N = 484 [61.7%] females vs. N = 300 [38.3%] males) (Chi2 = 43.2, df = 1; p = <0.001) and significantly more males in the SZ sample (N = 1.073 [64.4%] males vs. N = 594 [35.6%] females) (Chi2 = 137.6, df = 1; p = <0.001). The mean age at diagnosis of BD was 27.8 (SD = 4.0) years and it was 26.3 (SD = 4.1) years in the SZ sample. Age at first time diagnosis differed significantly between patients diagnosed with BD or SZ (Z = -8.6; p < 0.001).

In the two cohorts, N = 543 (69.3%) patients diagnosed with BD and N = 1,277 (76.6%) patients diagnosed with SZ had a preceding diagnosis. Lifetime preceding psychiatric disorders and preceding psychiatric disorders with onset in childhood and adolescence are shown in Table 1. Lifetime prevalence rates were high for preceding SUD (26.9%) and psychoses (41.5%) in SZ and for preceding affective disorders (46.6%) in BD. All prevalence rates of preceding disorders including ADHD and CD in particular were low during childhood and adolescence in both samples.

**Table 1:** Lifetime preceding psychiatric disorders and preceding psychiatric disorders with onset in childhood and adolescence in BD and SZ

	***Lifetime preceding psychiatric disorders***						***Preceding psychiatric disorders with onset before age 18***					
	**BD**		**SZ**				**BD**		**SZ**			
	**N**	**%**	**N**	**%**	**x2**	**p**	**N**	**%**	**N**	**%**	**x2**	**p**
Substance use disorders	126	16.1	449	26.9	35.0	<.001	9	1.1	26	1.6	0.6	.423
*Alcohol*	60	7.7	136	8.2	0.2	.667	1	0.1	3	0.2	0.0	.764
*Cannabis*	48	6.1	271	16.3	48.4	<.001	2	0.3	18	1.1	4.5	.034
*Opiods*	9	1.1	33	2.0	2.2	.139	0	0	0	0	-	-
Psychosis	111	14.2	691	41.5	180.4	<.001	14	1.8	35	2.1	0.3	.605
Affective disorders	365	46.6	466	28.0	82.3	<.001	15	1.9	46	2.8	1.6	.210
*Mania*	34	4.3	13	0.8	35.9	<.001	1	0.1	1	0.1	0.3	.585
*Depressive disorders*	325	41.5	450	27.0	51.6	<.001	14	1.8	42	2.5	1.3	.257
Anxiety disorders	69	8.8	187	11.2	3.3	.068	5	0.6	14	0.8	0.3	.595
Reactions to severe stress	206	26.3	444	26.6	0.0	.851	23	2.9	62	3.7	1.0	.321
*Acute stress reaction*	30	3.8	47	2.8	1.8	.182	2	0.3	3	0.2	0.1	.701
*PTSD*	10	1.3	41	2.5	3.7	.055	0	0	1	0.1	0.5	.493
*Adjustment disorder*	149	19.0	307	18.4	0.1	.727	17	2.2	48	2.9	1.0	.307
Personality disorders	171	21.8	374	22.4	0.1	.729	13	1.7	39	2.3	1.2	.275
*Paranoid*	4	0.5	10	0.6	0.1	.783	0	0	0	0	-	-
*Skizoid*	2	0.3	26	1.6	8.0	.005	0	0	1	0.1	0.5	.493
*Antisocial*	6	0.8	34	2.0	5.4	.020	2	0.3	5	0.3	0.0	.846
*Borderline*	87	10.7	159	9.5	0.8	.363	6	0.8	15	0.9	0.1	.736
ADHD	33	4.2	56	3.5	0.8	.373	9	1.1	12	0.7	1.2	.283
Conduct disorder	13	1.7	52	3.1	4.4	.036	9	1.1	34	2.0	2.5	.117
Any preceding disorder	543	69.3	1277	76.6	15.0	<.001	67	8.5	176	10.6	2.4	.120

BD = bipolar disorders; SZ = schizophrenia; ADHD = attention deficit hyperactivity disorder.

As seen in the lifetime frequencies in Table 1, preceding SUD in particular, due to cannabis use disorders, psychoses, schizoid and antisocial personality disorders, and CD were observed significantly more in SZ patients. Affective disorders and, in particular, mania and depressive disorders were significantly more frequent in BD patients. However, there was little differentiation of the two samples in terms of preceding disorders originating in childhood and adolescence. Only cannabis use disorder was significantly more common in SZ than in BD in this period.

Comparisons of the age of first time diagnosis of the preceding disorder for the major groups of disorders are shown in Table 2. The findings indicate that SZ was associated with a significantly earlier onset of preceding affective disorders, reactions to severe stress, and personality disorders.

**Table 2:** Age at first time diagnosis of preceding disorders in BD and SZ

	**BD**				**SZ**					
	**Mean**	**Median**	**SD**	**Range**	**Mean**	**Median**	**SD**	**Range**	**z**	**p**
Substance use disorders	24.3	24.0	4.9	[14;33]	23.4	22.0	4.9	[14;33]	- 1.8	.067
Psychosis	23.5	23	4.9	[14;34]	24.3	24.0	4.6	[5;34]	- 1.8	.076
Affective disorders	25.0	25.0	4.5	[13;34]	23.4	23.0	4.7	[11;34]	- 4.9	<.001
Anxiety disorders	24.1	24.0	4.6	[11;34]	23.2	23.0	4.5	[6;34]	- 1.5	.134
Reactions to severe stress	23.5	23.0	5.1	[10;34]	22.6	22	5.0	[9;34]	- 2.2	.027
Personality disorders	23.5	24.0	4.6	[12;34]	22.6	22.0	4.4	[12;34]	- 2.1	.035
Conduct disorder	18.9	16.0	7.1	[11;32]	15.6	15.0	6.4	[5;30]	-1.4	.168
ADHD	21.0	22	8.4	[3;33]	22.2	23.0	7.1	[5;33]	- 0.6	.530
Conduct disorder	18.9	16.0	7.1	[11;32]	16.0	16.0	6.3	[5;30]	-1.1	.278
Age at first preceding diagnosis	23.3	23.0	5.2	[3;34]	22.5	22.0	5.1	[5;34]	-3.2	.001

BD = bipolar disorders; SZ = schizophrenia; ADHD = attention deficit hyperactivity disorder.

 The results of the effect of birth year and sex on the risk of having the various preceding diagnoses are presented in Table 3, and the effect of the various comorbid disorders on the risk of BD/SZ is presented in Table 4. Male sex was a risk factor for SUD, psychosis and ADHD, while female sex was a risk factor for affective disorders, anxiety disorders, reactions to severe stress and personality disorders. There were significant effects on the risk of the preceding disorders associated with year of birth with persons born after 1980 having an elevated risk of being diagnosed with all psychiatric disorders. Most pronounced was the effect for ADHD and conduct disorder when controlling for length of follow-up time (Table 3). The adjusted models showed that ADHD was weakly associated with an increased risk for BD compared to SZ (Table 4). Preceding affective disorders were strongly associated with BD whereas SUD, psychoses, anxiety disorders and personality disorders increased the risk of SZ compared to BD. No differences were found for risk of diagnosis of BD compared to SZ for reactions to severe stress and conduct disorder.

**Table 3:** Sex and birth cohort as predictive for the risk of various preceding comorbid disorders in patients with Bipolar disorder/Schizophrenia

	Risk associated with sex*			Risk associated with birth cohort**		
	**OR**	**CI95%**	**p-value**	**HR**	**CI95%**	**p-value**
Substance use disorders	3.2	2.6 - 3.9	<.001	2.4	2.0 - 3.0	<.001
Psychoses	1.5	1.3 - 1.8	<.001	3.3	2.8 - 4.0	<.001
Affective disorders	0.4	0.4 - 0.5	<.001	3.2	2.7 - 3.8	<.001
Anxiety disorders	0.6	0.5 - 0.8	<.001	2.9	2.2 - 4.0	<.001
Reactions to stress	0.6	0.5 - 0.7	<.001	3.2	2.6 - 3.9	<.001
Personality disorders	0.6	0.4 - 0.6	<.001	1.8	1.5 - 2.2	<.001
CD	1.5	0.9 - 2.4	.159	11.3	4.1 - 31.1	<.001
ADHD	2.4	1.5 - 3.9	<.001	7.4	3.7 - 14.9	<.001

* Reference group: females. ** Reference group: persons born before 1980. HR= Hazard Ratio, OR= Odds Ratio.

## Discussion

First, preceding psychoses and SUD differed for BD and SZ. These disorders were associated with later SZ compared to BD patients, even after controlling for sex which is a known confounder for both psychoses and SUD. These findings are in line with expectations as psychoses are known to be precursors for SZ and SUD have been documented as a risk factor for psychoses. Therefore they are inherently also a risk factor for SZ [23,24,31]. Although psychoses and SUD have been documented as frequently occurring disorders in patients developing BD or SZ [21,22,26,27,29] in the present study the association was stronger for SZ and thus, may serve as evidence for differentiating preceding psychiatric histories of patients with BD or SZ.

**Table 4:** Comorbid disorders as predictors for Schizophrenia/Bipolar disorder

	**SZ**		**BD**			**SZ**		**BD**		
	***Crude***					Adjusted*				
	**OR**	**CI 95%**	**OR**	**CI 95%**	**p-value**	**OR**	**CI 95%**	**OR**	**CI 95%**	**p-value**
Substance use disorders	1.9	1.5 - 2.4	0.5	0.4 - 0.6	<.001	1.6	1.3 - 2.0	0.6	0.5 - 0.8	<.001
Psychoses	4.3	3.4 - 5.4	0.3	0.2 - 0.3	<.001	4.5	3.6 - 5.7	0.2	0.2 - 0.3	<.001
Affective disorders	0.4	0.4 - 0.5	2.2	1.8 - 2.7	<.001	0.5	0.4 - 0.7	1.9	1.5 - 2.2	<.001
Anxiety disorders	1.3	1.1 - 1.8	0.8	0.6 - 1.0	.069	1.5	1.1 - 2.0	0.7	0.5 - 0.9	.008
Reactions to stress	1.0	0.8 - 1.2	1.0	0.8 - 1.2	.851	1.2	1.0 - 1.4	0.9	0.7 - 1.1	.139
Personality disorders	1.0	0.8 - 1.3	1.0	0.8 - 1.2	.739	1.4	1.1 - 1.7	0.7	0.6 - 0.9	.003
CD	1.9	1.0 - 3.5	0.5	0.3 - 1.0	.039	1.4	0.8 - 2.7	0.7	0.4 - 1.3	.262
ADHD	0.8	0.5 - 1.3	1.2	0.8 - 1.9	.373	0.6	0.4 - 0.9	1.6	1.1 - 2.6	.033

* Adjusted for sex and age at BD/SZ diagnosis. OR= Odds Ratio.

Furthermore, after correcting for the confounding effect of the various co-variates included in the regression models, preceding anxiety disorders were also significantly associated with SZ with 11% of the affected patients having been diagnosed with an anxiety disorder preceding the first time diagnosis of SZ. This rate is lower than the findings of previous studies as indicated by a recent meta-analysis which calculated a prevalence rate of 38.3% for anxiety disorders in patients with SZ [18]. The prevalence rates for anxiety disorders in patients with BD were also lower in the present study than the findings from other studies [14,15]. Two major explanations may help to understand the divergence in findings. First of all, the present study solely reported anxiety disorders preceding SZ and BD and hence, the prevalence is expected to be lower than in studies where prevalence rates of anxiety were aggregated for concurrent and preceding diagnoses. Secondly, many anxiety disorders may have been treated in the primary sector by general practitioners as long as they were uncomplicated or not treatment- resistant. The present study only documented rates of anxiety disorders that were severe enough to have warranted referral to the psychiatric hospitals. When comparing the present findings to previous studies in terms of its differential impact of predicting SZ but not BD, there is some similarity to the findings from the Dunedin studies, which found that anxiety disorders were more frequent among patients who developed schizofreniform disorders whereas anxiety disorders were not associated with adult mania [1]. An increased risk for SZ compared to BD was also found for personality disorders. The nature of this finding is unclear. Future analyses might investigate how often the diagnoses of personality disorders are preceding diagnoses only and how many continue to be diagnosed as concurrent comorbid disorders even after the diagnoses of BD and SZ has been established.

Differences in the trajectories of preceding disorders of BD and SZ were found also for the affective disorders. Although affective disorders have frequently been documented in patients with SZ [20], they were a stronger predictor for BD than for SZ in the present study. This homotypic association may well have been the first episode of BD manifesting as depression before any episode of mania or hypo-mania had been diagnosed. The finding is in accordance with that from a US community study indicating continuity between adolescent depression and young adult BD [15] and the finding of the Dunedin birth cohort study indicating some continuity of juvenile depression and young adult mania [1].

Another preceding disorder that was more strongly associated with BD than SZ was ADHD, although the specific associations were less strong than the findings for affective disorders. Considering the recent debate in the research literature about whether BD in childhood might often be mistaken for ADHD [3], the rather low prevalence of 4.2% of patients with ADHD prior to BD in the present study is also noteworthy. This rate is just about equal to the prevalence of ADHD in some US community studies ranging between 1.9% and 5.7% for adolescents [4,8] and amounting to 4.4% in adults [5] but it is higher than in other studies [6-8]. If ADHD was a strong risk factor for BD, a higher prevalence should have been observed in the current study. Thus, the present finding is not in accordance with a review concluding that individuals with BD also seem to have high rates of ADHD [9], and a recent study finding high rates of concurrent and lifetime ADHD in a sample of BD patients using retrospective assessments, whereby 19% of the subjects screened positively for high rates of ADHD symptoms [32].

However, the nature of the present study cannot rule out the possibility that the association of BD and ADHD could have been stronger because there were time/cohort effects. Thus, it may well be that not all BD and SZ patients with ADHD had been identified and/or correctly diagnosed in the public mental health system in the total time period under investigation. Many cases in the study had been born, and were children in a time-period when ADHD was under recognized due to its non-specific diagnostic criteria in the ICD-8 classification for ADHD. Furthermore, as in most other countries, ADHD has only in the recent past become a disorder that is well recognized and diagnosed also in adults in Denmark.

 Although the literature documents an increased occurrence of CD in patients developing either BD or SZ as compared to the general population [14,17], the present study found no increase in risk for patients who developed BD in adulthood compared to those who developed SZ. As observed for ADHD, the prevalence rates for CD in both BD and SZ were lower than expected from previous studies that found preceding CD in patients with BD and SZ in the range of 21-43% [15-17]. However, cohort and time effects might have played a role in the present analysis.

In terms of the few similarities of the patterns of preceding disorders, reactions to stress was equally common in both samples of BD and SZ. Most probably, the reactions to stress reflect a generally heightened vulnerability of patients who later develop either BD or SZ.

In additional analyses, we looked at the manifestations of preceding disorders in childhood and adolescence and at the age at onset of these disorders. Whereas the rates of preceding disorders were quite frequent across the lifetime, they were rather low for all disorders originating in childhood and adolescence. In general, the mean age at onset was high, especially for disorders which are often diagnosed in childhood and early adolescence such as ADHD and CD. The mean age for first time diagnosis of any of the psychiatric disorders was 23 years for BD patients and 22.5 years for SZ patients. Preceding affective disorders, reactions to stress, and personality disorders were diagnosed earlier in SZ than BD and thus, may have contributed to an earlier vulnerability in SZ. The latter finding may be seen in line with the neurodevelopmental hypothesis of SZ assuming that both SZ and BD occupy a gradient with SZ reflecting a higher severity of neurodevelopmental impairment than BD [33].

The findings of both a relatively high age at first time diagnosis of any psychiatric disorders and of low frequencies of psychiatric disorders in childhood and adolescence do not necessarily indicate that a large number of disorders went unnoticed or unrecorded. They may also point to a sizeable number of subjects showing unclear preceding psychopathological phenomena in childhood and adolescence that may not exactly have met defining diagnostic criteria, but might later have been considered as prodromal features of a disorder. In addition, the results might also be explained by the before mentioned cohort/period effects. For all disorders, there were significant effect of year of birth with strongly increased hazard ratios starting with the year of birth in 1980 and onwards. This period matches the time when all preceding disorders in the lives of these patients had been diagnosed exclusively according to ICD-10-criteria which had been introduced in Denmark in 1994. Thus, classification effects may have been operating with the more detailed definition of disorders in the ICD-10 compared to the ICD-8 perhaps having led psychiatrists to diagnose an increasingly higher rate of disorders since the introduction of the ICD-10. Furthermore, the findings could be explained by the lack of registrations of outpatient contacts before 1995.

However, not only changes in the classification and registration may explain the findings of cohort-time effects, because incidence rates of various disorders have been found to increase in the ICD-10 period. This was found for e.g. depression, anxiety disorders, autism spectrum disorders and ADHD in a series of papers investigating the time trends in incidence rates of diagnosed mental disorders between 1995 and 2010 based on nationwide data-analysis using data from the DPCRR, by two of the authors of the present study. The observed increases in diagnosed incidence rates of mental disorders in the Danish population might have been both due to increases in the rates of psychiatric morbidity in the population and due to an increased awareness for mental disorders in both laymen and health care professionals leading to higher referral rates to psychiatry.

In terms of the clinical implications, it is noteworthy that a sizeable proportion of patients diagnosed in 2008–2010 for the first time with BD or SZ, namely 69.3% and 76.6%, had a preceding psychiatric diagnosis. In particular, the study presented additional evidence that patients with affective disorders should be thoroughly assessed for emerging symptoms of BD and patients with SUD and psychosis should be considered to be at risk for SZ. The study further documents that despite some shared genetic vulnerability, the developmental trajectories of BD and SZ are more different rather than similar in terms of preceding mental disorders.

Among limitations of the present study it has to be considered that all data stem from a register with a large number of clinicians from different institutions and regions contributing to the diagnoses over long time periods. First, all data reflect diagnoses as recorded in the psychiatric register implying that first manifestations of the disorder or sub-threshold symptoms of the disorder may have occurred earlier. Referral to and assessment within psychiatric services may have occurred later after patients experienced deficits in psychosocial functioning or had been in primary care before entering the psychiatric service system. Secondly, although the ICD-8/10 systems provides international comparability of diagnoses, the Danish data may to some extent reflect specific cultural attitudes towards using the various categories of the classification systems in clinical practice. However, these potential effects are largely unknown as long as there are no comparative studies based on large registers, which rarely exist outside the Scandinavian countries. Thus, the generalizability of the findings in the present study to other countries is difficult to evaluate. However, clinical assessments in Danish psychiatric hospitals are thorough and are completed over several assessment sessions by highly educated health care professionals in an interdisciplinary collaboration ensuring high quality of the psychiatric assessments.

Thirdly, as with all register studies the validity of the diagnoses is a critical issue. Various studies based on the DPCRR have shown that, in general, the validity of the diagnoses is high. This is documented for childhood autism [34], schizophrenia [35], and adult affective disorders [36]. So far, no controlled studies on the validity of ADHD and anxiety disorders in the DPCRR have been performed.

Even when considering these limitations, the relevance of the present findings should not be under-estimated since the alternative approach of prospective community studies analysing the preceding diagnoses of BD and SZ has not received sufficient research attention and/or is fraught with other limitations, including the size and representativeness of the sample. Furthermore, due to the rarity of both BD and SZ in the general population the course leading to the onset of these disorders is rather difficult to study prospectively in unselected populations.

## Conclusion

In the present study, two representative nationwide groups of patients with primary diagnoses of either BD or SZ in young adulthood were followed back to identify their preceding psychiatric diagnoses. The present study differs from all previous studies on comorbidity in BD and SZ by its emphasis on studying co-morbid mental disorders preceding the first time diagnosis of BD and SZ. A majority of patients diagnosed with BD and SZ had a psychiatric history preceding their first diagnosis of BD or SZ (69.3% and 76.6%) reflecting that both BD and SZ are serious mental disorders that gradually unfold. Furthermore, there were more differences rather than similarities in the patterns of preceding disorders of BD and SZ.

## Competing interests

H.–C. Steinhausen has worked as an advisor and speaker for the following pharmaceutical companies: Janssen-Cilag, Eli Lilly, Novartis, Medice, Shire, and UCB. In the past, he has received unrestricted grants for postgraduate training courses or conferences by Janssen-Cilag, Eli Lilly, Novartis, Medice, and Swedish Orphan International. Within the last four years, he has not received any financial support for drug studies. S. Andersen, A. Randers, C. Mohr Jensen, and C. Bisgaard, have no competing financial interest to disclose.

## Authors’ contributions

SMA and AR participated in the design and drafted the manuscript in cooperation with HCS and CMJ. CMJ and CB performed the statistical analysis of the data. All authors read and approved the final manuscript.

## Pre-publication history

The pre-publication history for this paper can be accessed here:

http://www.biomedcentral.com/1471-244X/13/343/prepub
